# Efficacy of Inhalable Endolysin Cpl-1 Formulations in Combination with Gentamicin or Endolysin Pal in a Murine Lung Infection Model

**DOI:** 10.1007/s11095-025-03942-z

**Published:** 2026-01-12

**Authors:** Yuncheng Wang, Maxwell T. Stevens, Trixie Wang, Adit B. Alreja, Daniel C. Nelson, Warwick J. Britton, Hak-Kim Chan

**Affiliations:** 1 Advanced Drug Delivery Group, School of Pharmacy, The University of Sydney, Sydney, NSW Australia; 2 Centre of Infection and Immunity, Centenary Institute, The University of Sydney, Sydney, NSW Australia; 3 Institute for Bioscience and Biotechnology Research, University of Maryland, Rockville, MD USA; 4 Department of Clinical Immunology, Royal Prince Alfred Hospital, Camperdown, NSW Australia

**Keywords:** endolysin Cpl-1 and Pal, gentamicin, inhalable formulation, lung infection, *Streptococcus pneumoniae*

## Abstract

**Purpose:**

Inhalable liquid formulation of endolysins represents a promising alternative to conventional antibiotics. Dry powder formulations offer improved stability for endolysin pulmonary delivery. This study aimed to evaluate the efficacy of an inhalable dry powder or liquid formulation of endolysin Cpl-1 alone and to compare it with liquid combinations of Cpl-1 with either gentamicin or endolysin Pal in a murine model of *S. pneumoniae* lung infection.

**Methods:**

A dry powder formulation of Cpl-1 was produced via spray drying, while liquid formulations were prepared by dissolving Cpl-1, or in combination with gentamicin or endolysin Pal in liquid. The droplet size distribution of aerosolized formulations was also characterized. Mice were intratracheally infected with *S. pneumoniae* and treated with either powder or liquid formulations. The bacterial load in respiratory system was assessed 26 h post-infection. The stability and activity of Cpl-1 in BALF were also evaluated *ex vivo*.

**Results:**

A single dose of Cpl-1 powder formulation or Cpl-1 liquid formulation (40 µg/mouse) reduced pulmonary bacterial load by approximately 1 log_10_. Importantly, the combination of Cpl-1 and Pal in liquid form resulted in a synergistic 2.0 log_10_ reduction, significantly greater than either endolysin alone, while combining Cpl-1 with gentamicin did not enhance antibacterial activity. *Ex vivo* assays confirmed that Cpl-1 retained full enzymatic activity after incubation in BALF.

**Conclusion:**

This proof-of-principle study demonstrated that inhalable endolysin liquid and powder formulations could potentially be used to treat bacterial lung infections. Moreover, the combination of multiple endolysins could increase antimicrobial activity over endolysin monotherapy.

## Introduction

It is widely recognized that bacteria, including *Streptococcus pneumoniae,* have increasingly developed resistance to antibiotics. Inhaled endolysin therapy has recently been investigated as an alternative for treating pulmonary bacterial infections [1]. Endolysins are bacteriophage-derived antibacterial proteins released at the terminal stage of the bacteriophage replication cycle that produce bacterial lysis. Unlike antibiotics, the potential resistance of bacteria to endolysins is considered to be much lower owing to their unique bactericidal mechanism targeting bacterial peptidoglycan [2].

Recent preclinical studies evaluating inhaled endolysin therapy have demonstrated promising results. In a murine *Staphylococcus aureus* lung infection model, intranasal delivery of endolysin SAL200 reduced bacterial load by ~ tenfold in the lungs and significantly increased the survival of mice as compared to PBS-treated mice [3]. In another study, two doses of endolysin PlyPa91 (either two intranasal treatments or one intranasal combined with one intratracheal treatment) decreased the mortality rate of *Pseudomonas aeruginosa*-infected mice, while the combined treatment resulted in higher survival rate of the mice (70% vs 20%) [4]. Our previous study showed that endolysin Cpl-1 could be aerosolized using a mesh nebulizer that resulted in retention of antimicrobial activity [5]. Doehn *et al*. confirmed that a high dose of Cpl-1 could rescue mice from fatal *S. pneumoniae* infection [1]. These studies focused on the efficacy of liquid preparations of endolysin because of the ease of formulation compared to dry powder preparations. Nevertheless, many studies have reported loss of activity of proteins in liquid formulations [6, 7], and this highlights the importance of testing endolysin powder formulations in *in vivo* infection studies.

Dry powder formulations offer several advantages over liquid formulations. Dry powder devices are more portable than nebulizers required for liquid formulation delivery. For example, tobramycin inhalable powder formulation can be readily inhaled without the need for electricity for aerosolization or a prolonged administration time, both of which are required for inhalation of tobramycin in solution [8, 9]. Furthermore, dry powder formulations show superior chemical stability compared to liquid protein formulations, and powders also avoid the liquid–air interface, which can cause protein degradation in liquid atomization [10]. We have recently developed a powder formulation of endolysin Cpl-1 with promising inhalation properties [11]. In the present study, this formulation has been used to test its antimicrobial effect in a mouse infection model.

Combination formulation is a strategy to enhance antimicrobial activity against bacterial infections through synergistic actions. A recent study demonstrated endolysin Cpl-1 in combination with gentamicin showed synergistic killing against the *S. pneumoniae* DCC1490 strain [12]*.* In another study, phage endolysins Cpl-1 and Pal exhibit a similar synergistic killing effect against *S. pneumoniae* DCC1490 [13]. Based on these findings, we developed liquid and powder formulations of endolysin Cpl-1, and its combination with gentamicin or endolysin Pal in liquid formulations, to treat *S. pneumoniae* lung infection.

The aim of this study is: 1) to determine the antimicrobial efficacy of the inhaled endolysin Cpl-1 powder and 2) to study the effect of liquid formulations of Cpl-1 in combination with endolysin Pal or gentamicin in a murine model of *S. pneumoniae* lung infection.

## Materials and Methods

### Bacteria and Active Pharmaceutical Ingredients

*S. pneumoniae* DCC1490 was obtained from the American Type Culture Collection and grown in tryptic soy broth supplemented with 15 g/L yeast extract at 37 °C. The genes for endolysin Cpl-1 and Pal inserted into the pET28b(+) vector with additional 6X-His tags, and then expressed in *E. coli* BL21(DE3) cells at 16°C with 0.2 mM isopropyl β-D-thiogalactoside induction. The recombinant proteins were purified using a nickel-nitrilotriacetic acid columns as previously described [14]. Purified proteins were dialyzed against phosphate-buffered saline (PBS), pH 7.4, concentrated, and lyophilized in 3 mg aliquots with 5% sucrose as the excipient. When required, the endolysin powders were dissolved and diluted to 400 μg/mL with distilled water. Protein concentrations were verified by assaying with the Pierce™ Coomassie Plus (Bradford) (Thermo Fisher Scientific, USA). Gentamicin was purchased from Sigma-Aldrich (Sydney, Australia).

### Liquid Formulations

Gentamicin powder was dissolved and diluted to 1.024 mg/mL with saline. The first combination formulation was made by mixing 1.6 mg/mL of endolysin Cpl-1 solution with 1.024 mg/mL of gentamicin solution. The second combination formulation was made by equally mixing 1.6 mg/mL of endolysin Cpl-1 solution with 1.6 mg/mL of endolysin Pal solution.

### Nebulization

Nebulization of Cpl-1, Pal and their combination solutions was conducted using a vibrating mesh nebulizer (eFlow Rapid, Pari Respiratory Equipment, USA). For each solution, 6 mL was nebulized and collected by condensation and coalescence into a test tube placed in an ice bath under vacuum with a pump maintained at 8 L/min [15] over the entire nebulization period.

### Aerosol Droplet Size Distribution

Aerosol droplet size distributions of Cpl-1, Pal and their combination liquid formulations were determined by laser diffraction (Spraytec Malvern Instruments Ltd., UK), as described previously [16]. Briefly, one-second measurements over 60 s of continuous nebulization was carried out for the mesh nebulizer. D_10_, D_50_ and D_90_ are the volume equivalent particle diameter at the 10^th^, 50^th^ and 90^th^ percentile of the particle population, respectively. The span of a volume-based size distribution is defined as (D_90_–D_10_)/D_50_, which indicates the distance between the 10 percent and 90 percent points, normalized with the midpoint of the particle diameter, i.e., the width of the size distribution. The fine particle fraction (FPF) represents droplets smaller than 5 μm, which are considered to be inhalable. The experiment was conducted in triplicate. Particle size distribution of the spray dried Cpl-1 powders (volume median diameter value of 1.5 ± 0.1 μm, span 1.5 ± 0.1) was previously reported [11].

### Powder Formulation Production By Spray Drying

Spray dried formulations of Cpl-1 and excipients was produced by spray drying using a spray dryer B-290 (Buchi Labortechnik AG, Switzerland) as described in [11]. Briefly, for the Cpl-1 powder, the liquid feed was composed of 2 mL of DI water or endolysin Cpl-1 solution (400 μg/mL) mixed with 2 mL of excipient solution (3.92 mg/mL leucine and 15.68 mg/mL trehalose), which means the final concentration of Cpl-1 in powder is 40 μg/2 mg. For excipient powder, 2 mL of DI water was mixed with 18 mL of excipient solution (4 mg/mL leucine and 16 mg/mL trehalose). A conventional two fluid nozzle was used for atomization. The solutions were fed at a feed rate of 1.9 mL/min, an atomizing airflow of 742 L/h and an aspiration rate of 35 m^3^/h. The inlet temperature was 60 °C and the outlet temperature was between 40 and 42 °C. Powder was collected in a vial and stored with silica beads at room temperature before use.

### Animal Experiments

All animal experiments were approved by Sydney Local Health District Animal Welfare Committee (Sydney, NSW, Australia); approval number 2021/011. Female, BALB/c, 6–8 week-old mice were purchased from Australian BioResources (Moss Vale, NSW, Australia) and kept in a specific pathogen-free environment at Centenary Institute (Camperdown, NSW, Australia), with water and food readily available. On the day of inoculation (day 0), mice (4 mice per group) were anesthetized by intraperitoneal injection (80 mg/kg ketamine and 8 mg/kg xylazine in PBS). With an otoscope, 25 μL PBS (control group) or bacteria suspension (approximately 2 × 10^5^ CFU) were visually administered into the trachea with Penn-Century MicroSprayer^®^ Aerosolizer. Antibacterial treatments were given 2 h after inoculation with mice anesthetized by intraperitoneal injection (80 mg/kg ketamine and 8 mg/kg xylazine in PBS) again. Twenty-five μL of liquid formulations (Table I) were visually administered into the trachea with a Penn-Century MicroSprayer^®^ Aerosolizer. For dry powder formulation, 2 mg excipient of Cpl-1 powder (containing 40 μg of Cpl-1), or 0.5 mg Cpl-1 powder (containing 10 μg of Cpl-1) was loaded into a 200-μL gel-loading pipette tip and then expelled with 0.4 mL air using a 1 mL syringe linked with a three-way valve as described previously [17]. Mice were euthanized by CO_2_ asphyxiation at 26 h post-inoculation. To ensure collection of bacteria in the airways, bronchoalveolar lavage fluid (BALF) was obtained by instilling 1 mL of PBS through the trachea to the lung with a 1-mL syringe and collecting the lavage suspension. Afterward, the whole lung was also collected and homogenized in PBS with a Polytron PT10-35 homogenizer connected to PCU power control unit (Kinematica AG Littau/Luzern, Switzerland). The lung homogenate and BALF were mixed for CFU determination (see Sect. "Colony counting:").

**Table I:** Liquid Formulations used for Intratracheal Treatments

Formulations	1	2	3	4	5	6
Component(s) dispersed in 25 μL of PBS (/mouse)	Cpl-1 40 μg	Gentamicin 25.6 μg	Pal 40 μg	Cpl-1 20 μg, Gentamicin 12.8 μg	Cpl-1 20 μg, Pal 20 μg	PBS control

### Activity of Endolysin Cpl-1 in Bronchial Alveolar Lavage Fluid

Endolysin Cpl-1 powder was dissolved to 80 μg/mL in water, as confirmed by protein concentration assay measured with the BCA kit (Thermo Fisher Scientific, USA). To prepare the Cpl-1 and BALF sample, 1 mL of endolysin Cpl-1 solution was mixed with either 1 mL of PBS or BALF collected from anesthetized healthy mice (as in Sect. "Animal experiments" above) followed by 24 h incubation at 37℃ with shaking at 200 rpm. A single colony of *S. pneumoniae* DCC1490 was cultured overnight in 20 mL tryptic soy broth supplemented with 15 g/L yeast extract. This culture was centrifuged at 3000 rpm using Beckman Coulter Allegra X-12R Centrifuge (Pasadena, CA, United States) at a temperature of 4 °C for 10 min and washed three times with PBS, then diluted to an OD600 of 0.8–0.1 in PBS to prepare the bacterial stock. OD600 was measured using a POLARstar Omega microplate reader (BMG Labtech, Germany). In a 96-well plate, 100 μL of PBS, Cpl-1 in PBS, and Cpl-1 in BALF samples were mixed with 100 μL of bacterial stock, and the mixed solutions were incubated for 10 min at 37 °C, before plating on sheep blood agar plates for colony counting (n = 3 independent biological replicates).

### Colony Counting

Samples from animal experiments and Cpl-1 activity test in BALF and lung homogenate from healthy mice were serially diluted (1:10) in PBS and plated on sheep blood agar plates followed by incubation at 37 ℃ for 24 h. CFU counts were then determined as described previously [18].

### Statistics

Statistical tests were conducted using Prism software version 10 (GraphPad Software Inc., San Diego, CA, USA) and analyzed by one-way analysis of variance (ANOVA) followed by post hoc Tukey’s test.

## Results

### Aerosol Droplet Size Distribution

The D_50_ of droplets produced by the mesh nebulized samples were 4.2 ± 0.2 µm (span 1.2–1.4), 4.2 ± 0.1 (span 1.1–1.3) and 3.9 ± 0.1 µm (span 1.1–1.2) for Cpl-1, Pal and their combination liquid formulation. The differences in the size of droplets generated were not statistically significant (p ≥ 0.05). The FPF values were 61.9 ± 2.1%, 65.0 ± 2.6% and 64.3 ± 4.6% for the mesh-nebulized Cpl-1, Pal, and their combination, respectively, showing no significant differences (p > 0.05). The D_50_ of all liquid formulations were smaller than 5 μm in diameter, a size which is considered inhalable [19], and is consistent with our previous study [5].

### *Ex vivo* Activity Loss Of Endolysin Cpl-1 Powder Formulation In Healthy Mice’s BALF

**Fig. 1:**
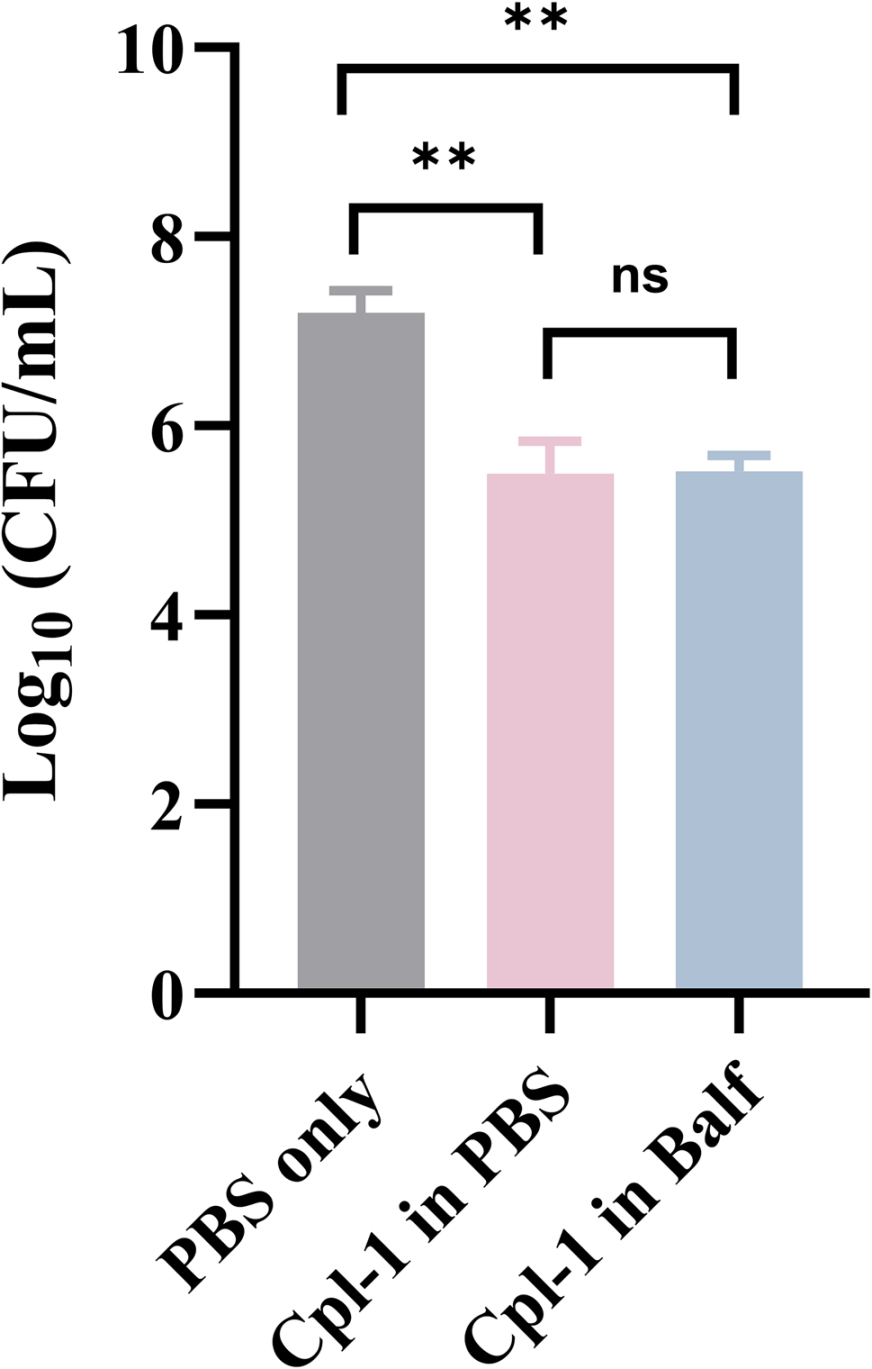
The antimicrobial activities of endolysin Cpl-1 powder against *S. pneumoniae* DCC1490 before and after incubation with BALF. Data are shown as the mean CFU/mL ± standard deviation (*n* = 3). The statistical differences between groups were tested by ANOVA; **, *p* < 0.01; ns, not significant.

The enzyme, both with and without exposure to BALF, demonstrated statistically significant antimicrobial activity compared to PBS controls (showing one-fold increase in killing for each condition). However, there was no statistically significant difference observed between the enzyme exposed to BALF and the enzyme incubated with PBS (Fig. 1). This finding suggests that proteases, other cellular factors, and immune cells within the BALF did not inactivate or degrade Cpl-1, thereby supporting the translational use of this enzyme in the lung environment. This confirmed that endolysin Cpl-1 retains its activity in BALF and is suitable for lung delivery in mice.

### Activity of Endolysin Cpl-1 Powder Formulations Against *S. pneumoniae* Infection *In Vivo*

**Fig. 2:**
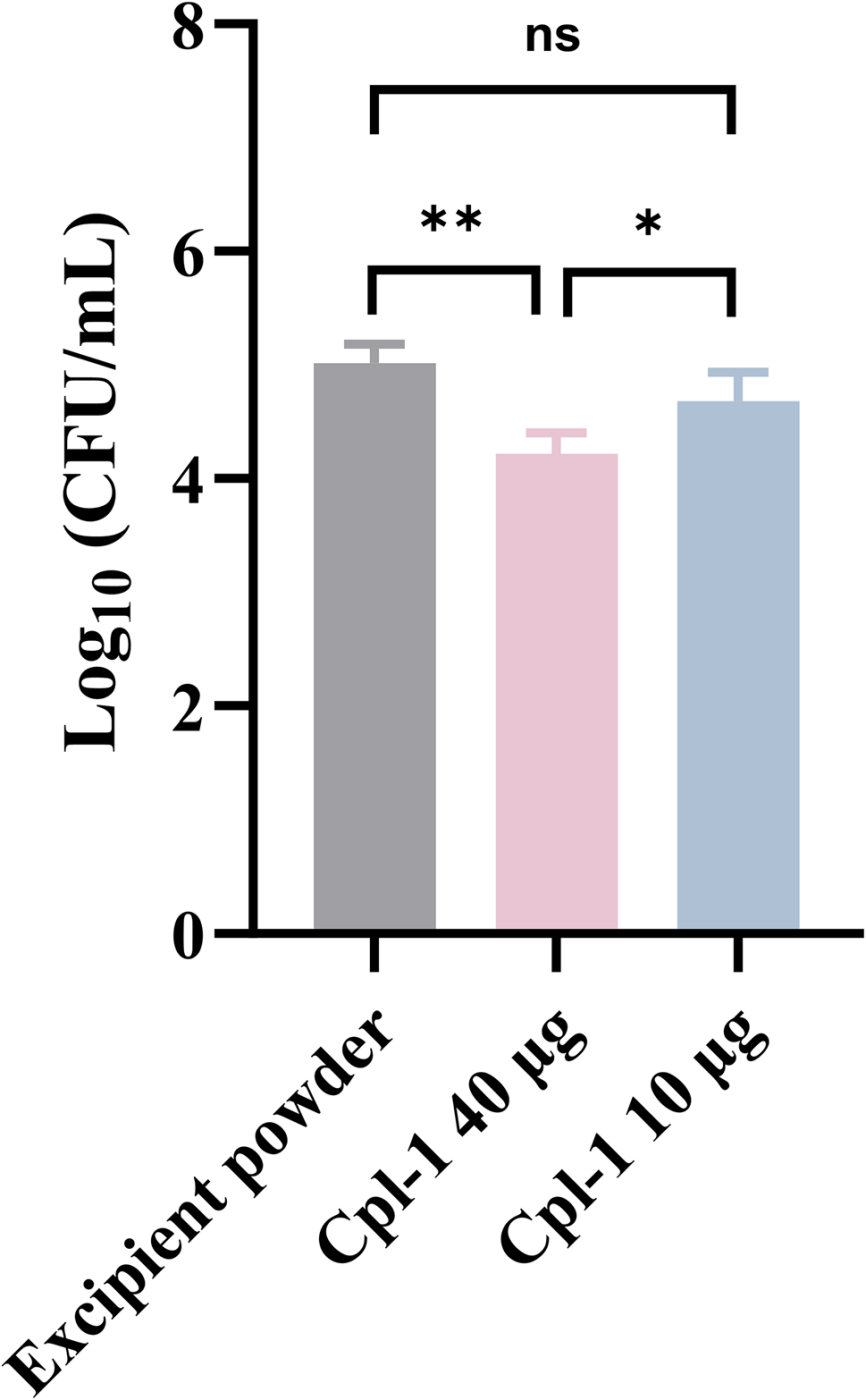
The antimicrobial activities of endolysin Cpl-1 powder at different doses against *S. pneumoniae* DCC1490 infection in lung infection model in BALB/c mice. Data are shown as mean CFU/mL in lung homogenates and BALFs ± standard deviation (*n* = 4). The statistical differences between groups were tested by ANOVA; *, *p* < 0.05; **, *p* < 0.01; ns, not significant.

We previously established that spray-dried endolysin Cpl-1 retained its antibacterial activity *in vitro* after 2 months' storage [11], and now examined the activity of the dry powder formulation in the *in vivo* model of *S. pneumoniae* infection. The Cpl-1 powder at the higher dose of 40 µg/mouse significantly decreased the pulmonary bacterial load by approximately 0.9 log_10_, while the dose 10 µg/mouse of Cpl-1 powder did not reduce the bacterial load (Fig. 2). This indicates that the antimicrobial activity of Cpl-1 in powder is dose-dependent. Due to the limited supply of the endolysin, although dose higher than 40 µg/mouse was not used, it is sufficient for the purpose of this proof-of-concept study based on the powder prepared previously [11].

### Activity of Endolysin Cpl-1 In Combination With Endolysin Pal or Gentamicin Against *S. pneumoniae* Infection *In Vivo*

**Fig. 3:**
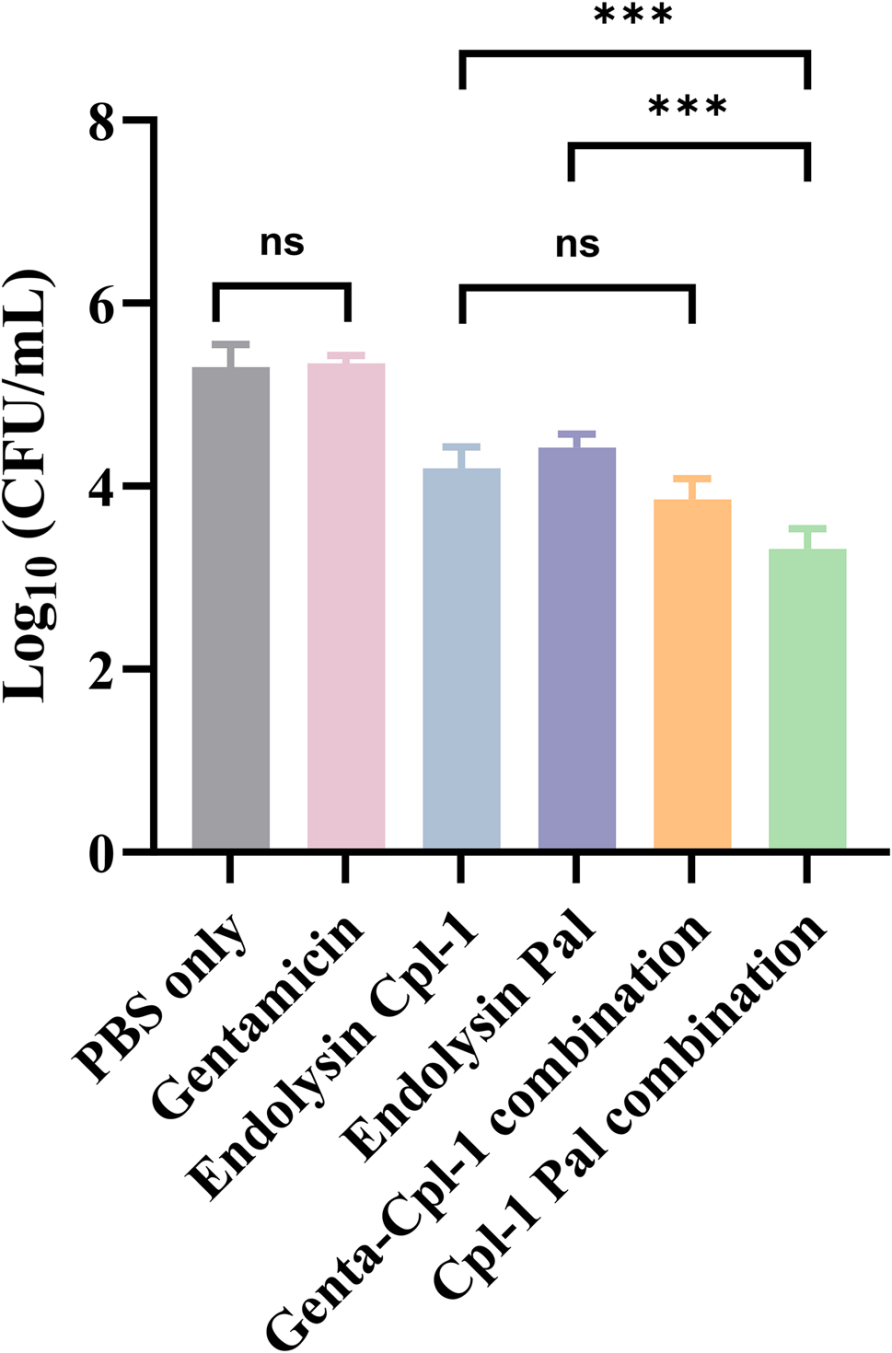
Activity of endolysin Cpl-1 (40 μg), endolysin Pal (40 μg), gentamicin (25.6 μg), or their combinations (12.8 μg of gentamicin with 20 μg of Cpl-1, 20 μg of endolysin Pal with 20 μg of Cpl-1) against *Streptococcus pneumoniae* DCC1490 strain in a murine lung infection model. Data are shown as mean CFU/mL ± standard deviation (*n* = 4). All groups except gentamicin only group showed significant differences with PBS only group. *, *p* < 0.05; ***, *p* < 0.001; ns, not significant.

The activity of a liquid formulation of endolysin Cpl-1 alone or in combination with endolysin Pal or gentamicin was examined in *S. pneumoniae* infection in mice. Single treatment of Cpl-1 liquid formulation decreased the murine pulmonary bacterial load by approximately 1.1 log_10_ and endolysin Pal had a similar effect (Fig. 3). By contrast, the combination of endolysin Cpl-1 and endolysin Pal caused a significantly greater reduction of approximately 2 log_10_ in bacterial load. Treatment with gentamicin alone had no activity in this model, and there was no significant difference in bactericidal efficacy between the mice receiving gentamicin/endolysin Cpl-1 and the Cpl-1 alone. Therefore, the combination of two endolysins had the largest antibacterial effect *in vivo*.

## Discussion

This is the first study to assess the *in vivo* activity of inhalable formulations of endolysin Cpl-1 and its combinations with an antibiotic or a different endolysin against *S. pneumoniae*. We evaluated the anti-*Streptococcus* endolysin Cpl-1 in both liquid and powder formulations.

Our previous study demonstrated the potential for nebulization of Cpl-1 liquid formulations for lung delivery [5]. This current study showed that the aerosol particle size distribution and FPF values of the formulations were suitable for efficient delivery of the protein. In general, delivery of therapeutic agents to the lungs requires particle size of 5 µm or less [19]. The D_50_ values of the aerosols are below this size and the span values show the aerosols are mildly to moderately polydisperse, suggesting they will deposit in various parts of the respiratory tract. Endolysin Cpl-1 (400 μg/mL) in spray drying liquid feed containing excipients (4 mg/mL of leucine, 20 μg/mL of sucrose, 16 mg/mL of lactose or trehalose) started to form aggregates after three days of storage at room temperature as previous reported [11], which is possibly due to surface denaturation of Cpl-1 molecules coming into contact with the air–liquid and liquid–solid interfaces in the falcon tube. It is well known that proteins can spontaneously adsorb onto a wide variety of surfaces. In particular, liquid interfaces allow adsorbate molecules a higher degree of mobility along the interface to penetrate into the non-aqueous phase such as air to cause unfolding of the protein molecules followed by aggregation, e.g., hydrophobic interactions [20]. Future work is necessary to confirm or refute this hypothesis. This will include formulation approaches using various stabilisers such as saccharides, polyethylene glycols and divalent cations [21]. In contrast, the endolysin Cpl-1 powder formulation possessed excellent inhalation properties and retained antimicrobial activity after 2 months [11]. This highlighted the value of developing dry powder formulations of endolysins. Although a comprehensive stability study of various formulations was not feasible due to the limited supply of Cpl-1, previous work of the Cpl-1 powder formulation by the authors has provided data on in-depth physical and chemical characterization, confirming retention of the activity of endolysin Cpl-1, and promising inhalation performance of the powder [11].

Doehn *et al*. found that intratracheal delivery of 400 μg/mouse of Cpl-1 in liquid successfully decreased the *S. pneumoniae* bacterial load in the lung by approximately 100-fold 24 h after treatment in a murine lung infection model as compared to mice treated with PBS [1]. However, for the dry powder delivery, murine tracheal tolerance limits the maximum dose for one mouse to 2 mg of powder, which confined the highest dose of Cpl-1 powder used in this study to 40 μg/mouse [22]. Therefore in powder formulation 1 (Table II), which is based on our previous reported Cpl-1 powder formulations [11], we used 2 mg (containing 40 μg Cpl-1) per mouse for the efficacy study. To compare the efficacy of powder and liquid formulations, we used the same amount of Cpl-1 (40 μg/mouse) in liquid formulation 1 (Table I). The amounts of gentamicin and Pal in formulations 2 and 3 are calculated based on the concentration ratios of Cpl-1/gentamicin and Cpl-1/Pal in *in vitro* synergy studies reported in references [12] and [13]. In formulations 4 and 5, we mixed two single-drug formulations in equal proportions for animal study. As a result, the concentration of each individual drug in the same volume of liquid was reduced to half of the original level. This ratio is also consistent with those used in references [12] and [13]. As we used lower doses (40 μg and 10 μg per mouse) of Cpl-1 in our powder formulation than that in the Doehn study, we examined an earlier treatment time (2 h post infection compared to 24 h post infection) to test the efficacy of Cpl-1 powder *in vivo*.

**Table II:** Dosage of Cpl-1 Powder Formulations for Intratracheal Treatments

Formulations	1	2	3
Dry powder containing (/mouse)	Cpl-1 40 μg	Cpl-1 10 μg	Only excipients*

^*^Excipient concentration used was 4 mg/mL leucine and 16 mg/mL trehalose

We assessed the activity of endolysin Cpl-1 in the BALF from healthy mice prior to the *in vivo* test. The results confirmed that endolysin Cpl-1 retained its full activity in diluted murine BALF even after 24 h of co-incubation (Fig. 1). This finding suggests that proteases, other cellular factors, and immune cells within the BALF did not inactivate or degrade Cpl-1, thereby supporting the translational use of this enzyme in the lung environment.

A single treatment of Cpl-1 decreased the pulmonary bacterial load by 0.9–1.1 log_10_ in mice that received 40 μg of Cpl-1 in liquid or powder formulations (Fig. 2 and Fig. 3), which confirmed the spray-dried form retained antimicrobial activity of Cpl-1. However, a lower dose of 10 μg of Cpl-1 in powder caused no significant reduction of bacterial load in murine lungs compared with controls, indicating the dose-dependent efficacy of the inhalable Cpl-1 powder.

Although a single dose of Cpl-1 in liquid or powder form reduces the bacterial load by approximately 0.9–1.1 log_10_, achieving a stronger bactericidal effect may be necessary for effectively treating bacterial infections in the lungs. Therefore, we evaluated combination therapies of Cpl-1 with gentamicin, or Cpl-1 with another endolysin, Pal, in liquid formulations. Cpl-1 was reported to have synergistic effect with gentamicin and endolysin Pal against *S. pneumoniae* strain DCC1490 in both checkerboard and time-kill assays [12]. The MIC values of gentamicin and Cpl-1 for *S. pneumoniae* were 16 μg/mL and 25 μg/mL, respectively. Our results show that a half dose of Cpl-1 (20 μg/mouse) combined with a half dose of gentamicin (12.8 μg/mouse) did not result in a significantly greater bacterial load reduction compared to single treatments of Cpl-1 (1.1 log_10_ bacterial load reduction) or gentamicin that was not effective at this dose. This may be due to the fact that the fractional inhibitory concentration index (FICI) of gentamicin and Cpl-1 is only 0.5 [12], indicating weak synergy (FICI values of 0.5 or less are considered to show synergistic effects). In contrast, a half dose of Cpl-1 (20 μg/mouse) in combination with a half dose of Pal (20 μg/mouse) showed a higher antimicrobial activity with a 2.0 log_10_ bacterial load reduction, compared with the single treatment of Cpl-1 (1.1 log_10_ bacterial load reduction) or Pal (0.9 log reduction) at twice the dose (40 μg/mouse).

In this proof-of-concept study, we confirmed that endolysin Cpl-1 in powder could maintain its antimicrobial activity in BALF *ex vivo* over at least a 24 h period, and lead to a dose-dependent decreased bacterial load in mice with a *S. pneumoniae* lung infection. In addition, the combination of endolysin Cpl-1 and endolysin Pal in liquid showed a significant higher antibacterial activity compared with either endolysin Cpl-1 or endolysin Pal monotherapy. Future pharmacokinetic, pharmacodynamic, and toxicity studies are needed to continue the translational development of inhalable endolysin treatments.

## Conclusion

We present the first proof-of-principle study investigating the efficacy of endolysin Cpl-1 dry powder and its combination formulations in liquid containing gentamicin or Pal in a murine *S. pneumoniae* lung infection model. The results showed that these formulations could potentially be used to treat *S. pneumoniae* lung infections.

## Data Availability

The datasets generated during and/or analysed during the current study are available from the corresponding author on reasonable request.
